# The Association Between Peptic Ulcer Disease and Ischemic Stroke: A Population-Based Longitudinal Study

**DOI:** 10.1097/MD.0000000000003797

**Published:** 2016-06-03

**Authors:** Tain-Junn Cheng, How-Ran Guo, Chia-Yu Chang, Shih-Feng Weng, Pi-I Li, Jhi-Joung Wang, Wen-Shiann Wu

**Affiliations:** From the Department of Neurology (T-JC, C-YC); Department of Occupational Medicine (T-JC), Chi Mei Medical Center, Yongkang; Department of Occupational Safety and Disaster Prevention (T-JC), College of Sustainable Environment, Chia Nan University of Pharmacy and Science, Jen-Te; Department of Environmental and Occupational Health (H-RG, P-IL), College of Medicine; Department of Occupational and Environmental Medicine (H-RG), National Cheng Kung University Hospital; Center for General Education (C-YC), Southern Taiwan University, Yongkang, Tainan; Department of Healthcare Administration and Medical Informatics (S-FW), Kaohsiung Medical University, Kaohsiung; Department of Family Medicine (P-IL); Department of Medical Research (J-JW); Department of Internal Medicine (W-SW), Chi Mei Medical Center; and Department of Pharmacy (W-SW), Chia Nan University of Pharmacy and Science, Jen-Te, Tainan, Taiwan.

## Abstract

Stroke is a common cause of death worldwide, but about 30% of ischemic stroke (IS) patients have no identifiable contributing risk factors. Because peptic ulcer disease (PUD) and vascular events share some common risk factors, we conducted a population-based study to evaluate the association between PUD and IS.

We followed up a representative sample of 1 million residents of Taiwan using the National Health Insurance Research Database from 1997 to 2011. We defined patients who received medications for PUD and had related diagnosis codes as the PUD group, and a reference group matched by age and sex was sampled from those who did not have PUD. We also collected data on medical history and monthly income. The events of IS occurred after enrollment were compared between the 2 groups. The data were analyzed using Cox proportional hazard models at the 2-tailed significant level of 0.05.

The PUD group had higher income and prevalence of hypertension, diabetes mellitus (DM), heart disease, and hyperlipidemia. They also had a higher risk of developing IS with an adjusted hazard ratio of 1.31 (95% confidence interval: 1.20–1.41). Other independent risk factors included male sex, older age, lower income, and co-morbidity of hypertension, diabetes mellitus (DM), and heart disease.

PUD is a risk factor for IS, independent of conventional risk factors such as male sex, older age, lower income, and co-morbidity of hypertension, DM, and heart disease. Prevention strategies taking into account PUD should be developed and evaluated.

## INTRODUCTION

Ischemic stroke (IS) is a cerebrovascular disease (CVD) that often presents acute neurological deficits from abnormal vascular supply in the central nervous system and is a major cause of death (COD) in adults. Although the mortality rate of stroke declines worldwide, including in Taiwan,^1^ the prevalence and incidence of IS remain high in many countries. In the United States, CVD was the 2nd leading COD in the 1950s and was still ranked the 4th in 2011.^2^ In Taiwan, it has been among the top 3 leading CODs for the past 30 years.^1^ Additionally, it is a major health burden leading to a large proportion of adult disabilities, considerable national economic loss, and substantial social and financial impacts on patients and their families.^3^ Therefore, preventing CVD and decreasing the severity of deficits to minimize disability is an important task of clinicians and public health practitioners.

More than 300 risk factors have been reported for CVD, and about 75% of stroke events are associated with the conventional risk factors including aging, family history, male sex, hypertension, hyperlipidemia, diabetes mellitus (DM), tobacco, and physical inactivity.^4^ Over time, the mortality of CVD declined with the efforts of preventing risk factors and treating modifiable risk factors such as hypertension, DM, and hyperlipidemia. However, a considerable number of CVD patients have no identifiable causes. To prevent CVD, we should keep exploring unidentified risk factors.

Psychological stress can arise from acute stressors such as natural disasters, physical illness, and major traumas, as well as chronic stressors such as socioeconomic burdens, unpleasant family and unfriendly personal relationship, overload at work, and uncomfortable workplace. It can change the cortisone level and the balance of sympathetic and parasympathetic nervous activities, presenting physical manifestations such as changes in heart rate variability, dysrhythmias, and ventricular wall dysfunction.^5^ Although it is difficult to precisely define the meaning of psychological stress, there are abundant evidences from the literature demonstrating that it can cause cardiovascular disease (CAD). For example, an acute but reversible heart failure called Takotsubo cardiomyopathy can be caused by abnormal catecholamine dynamics soon after sudden, unexpected emotional or physical stress.^6^ In the Atherosclerosis Risk in Communities Study, normotensive participants with high anger temperament scores had a hazard ratio (HR) of 2.3 for developing cardiac events.^7^ The International Labor Office estimated that 21% of work-related mortality is related to circulatory diseases, with job stress as one of the major contributing causes.^8^ The Study of Women's Health Across the Nation showed that stress can increase the carotid intima thickness, which is a good indicator of atherosclerosis, a risk factor for CVD.^9^

Similarly, peptic ulcer disease (PUD) can be caused by both acute and chronic stressors^10^ and was associated with personality, life events, and psychosocial factors.^11^ Even though PUD is now considered mostly as an infectious disease associated with *Helicobacter pylori* (HP),^12^ it was estimated that 30% to 65% of PUD patients were related to psychosocial stress.^13^ Because psychological stress has long been regarded as a possible risk factor for IS^14,15^ and PUD is a good indicator of physical responses to psychological stress, we speculated that PUD may be a risk for IS and conducted a study to evaluate the possible association accordingly.

## METHODS

We conducted a population-based cohort study from 1997 to 2011 by analyzing the Longitudinal Health Insurance Database 2000 (LHID2000) of the National Health Insurance Research Database (NHIRD) of Taiwan, created by the National Health Insurance (NHI) program in 1996.^16^ The NHI program is a nationwide healthcare system in Taiwan established in 1995, and its coverage rate exceeded 99% in 2007. The LHID2000 consists of 1 million randomly sampled people corresponding to about 4% of all enrollees. There were no differences in demographic characteristics, including age, sex, and income, between the selected sample and all enrollees.^16^

The LHID2000 contains separate datasets, and the information on each member includes an encrypted personal identification number, sex, date of birth, diagnostic codes using the *International Classification of Diseases, Ninth Revision, Clinical Modification* (*ICD-9-CM*), drug prescriptions, medical cost, medical care facilities, and specialty of caregivers, both in outpatient and hospitalization settings. All datasets are interlinked by the personal identification number of the patient.^16^

### Study Population

**FIGURE 1:**
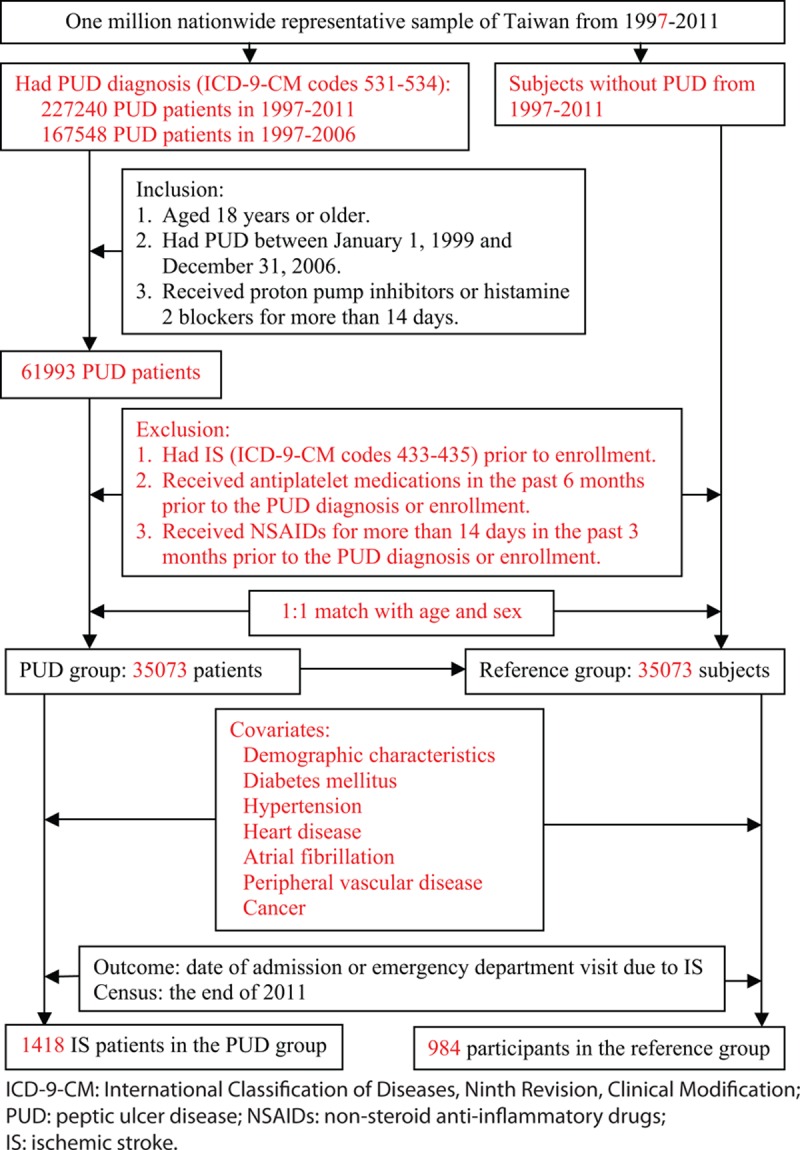
Study diagram.

For the PUD group, we selected adult patients (aged 18 years or older) with a primary diagnosis with *ICD-9-CM* codes 531 to 534 who received prescriptions of medication for PUD, including proton pump inhibitors (PPIs) and histamine 2 (H2) blockers. (Figure 1) To be qualified as a PUD case, the prescriptions must cover a duration of >14 days between January 1, 1999 and December 31, 2006. To avoid overdiagnosis of PUD, we did not enroll subjects with only either diagnosis codes or medications (PPI or H2 blocker) of PUD as PUD patients. All patients were verified for not having IS (*ICD-9-CM* codes 433–435) from January 1, 1997 to the date of enrollment. Patients who had received prescriptions of antiplatelet medications in the 6 months before PUD diagnosis or nonsteroid anti-inflammatory drug (NSAID) for >14 days in the 3 months before PUD diagnosis were excluded. All the subjects free of PUD from 1997 to 2011 were candidates of references. One reference each was randomly selected from the candidates who had the same sex and year of birth as a PUD patient. As in the PUD group, members of the reference group were free of IS and were not prescribed antiplatelet or NSAID medications. All participants were followed up until the date of admission or emergency department visit because of IS with the diagnostic code between 433 and 435, death, or the end of 2011, whichever was earlier.

On each member of the 2 study groups, we collected information including demographic characteristics, risk factors, and comorbidities coded using *ICD-9-CM*, including DM (250), hypertension (401–405), hyperlipidemia (272), heart diseases (410–429, except 427.3), atrial fibrillation (427.3), peripheral vascular disease (PVD) (440–459), and cancers (140–208). The heart diseases included ischemic heart disease (410–414), disease of pulmonary circulation (415–417), cardiomyopathy (425), conduction disease (426), cardiac dysrhythmia (427, except 427.3), heart failure (428), and other forms of heart disease (420–424, 429).

### Statistical Analysis

All the data processing and statistical analysis were performed by using SAS statistical software (SAS Institute Inc, Version 9.3.1, Cary, NC). *χ*^2^ Tests were used to evaluate the differences in categorical data, whereas Student *t* tests were used for continuous variables. Kaplan-Meier analyses were used to calculate the cumulative incidence of stroke in the 2 groups, and log-rank tests were used to evaluate the differences between PUD patients and references. The Cox regression models were constructed to evaluate effects of the risk factors for CVD, including PUD and the 4 comorbidities. A 2-tailed *P* value of <0.05 was considered statistically significant. This study was approved by the institutional review board of the Chi Mei Medical Center.

## RESULTS

The number of PUD patients identified with the diagnostic codes 531 to 534 was 227,240 between 1997 and 2011 and 167,548 between 1997 and 2006. There were 61,993 PUD patients who were prescribed with PPI or H2 from 1999 to 2006. After excluding previous diagnosis of IS and prescription of NSAID or antiplatelet medications, we enrolled 35,073 patients in the PUD group, which consisted of 20,214 (57.6%) men and 14,859 (42.4%) women with an average age of 50.9 years (standard deviation: 16.35 years). (Table 1) The PUD group had higher prevalence of DM (10.4% vs 6.3%), hypertension (8.3% vs 11.6%), heart disease (10.4% vs 4.8%), atrial fibrillation (0.6% vs 0.3%), PVD (4.0% vs 1.3%), hyperlipidemia (5.6% vs 3.1%), and cancer (2.4% vs 1.5%), all with *P* < 0.001.

**TABLE 1:**
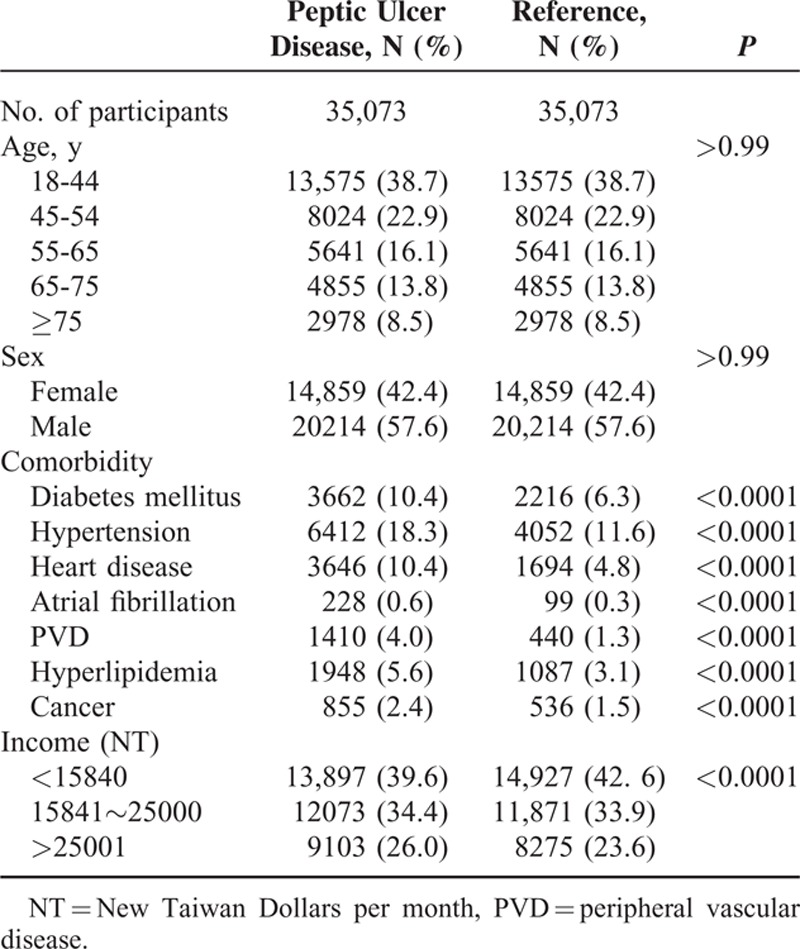
Demographic Characteristics and Comorbid Medical Disorders for Peptic Ulcer Disease and Reference Groups From 1997 to 2011

A total of 236509.8 person-years were followed with 1418 IS cases identified in the PUD group, and 243532.6 person-years were followed with 984 IS cases identified in the reference group. (Table 2) The incidence of IS was higher in the PUD group (60.0 vs 40.4 per 10,000 person-years) with a crude HR of 1.48 (95% confidence interval [CI]: 1.37–1.61). An increased risk of developing IS in the PUD patients was observed in all categories stratified by sex, age, and duration of follow-up. In addition, the crude HR increased with age (Table 3).

**TABLE 2:**
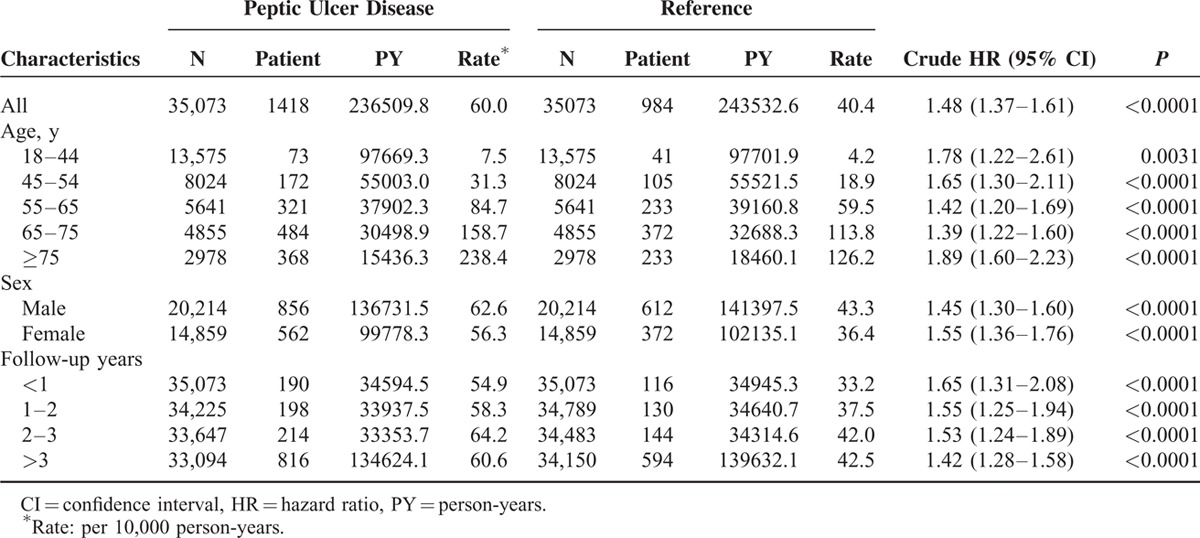
Risks of Developing Ischemic Stroke in the Peptic Ulcer Disease and Reference Groups From 1997 to 2011 in Taiwan

**TABLE 3:**
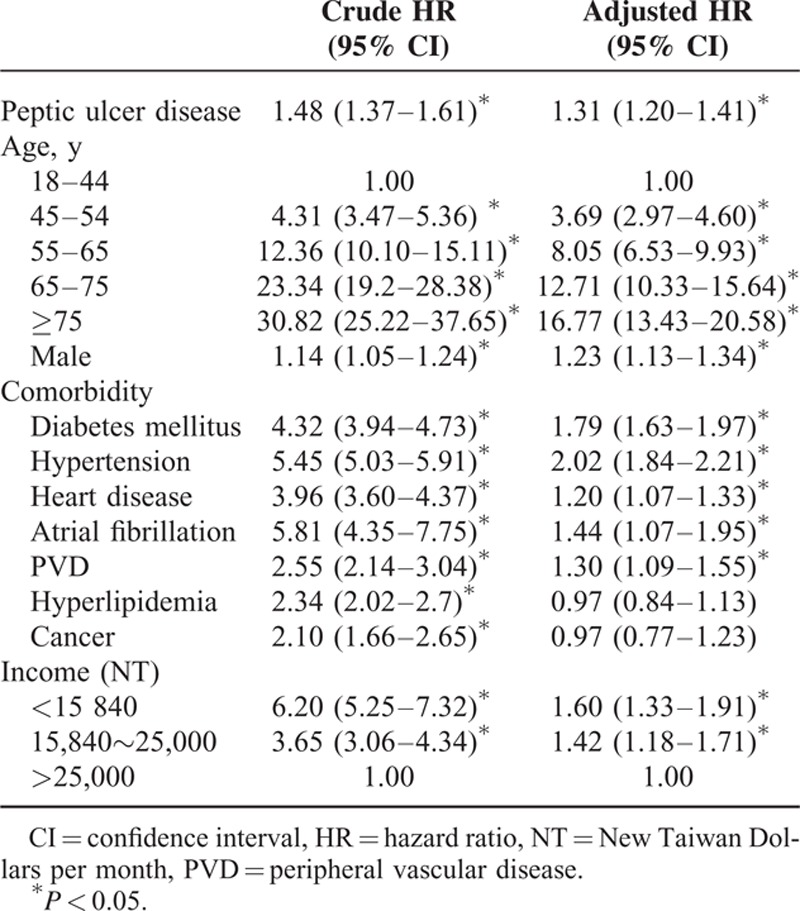
Crude and Adjusted HRs for the Development of Ischemic Stroke

In the univariate analyses, other risk factors for IS included male sex (crude HR = 1.14, 95% CI: 1.05–1.24), DM (crude HR = 4.32, 95% CI: 3.94–4.73), hypertension (crude HR = 5.45, 95% CI: 5.03–5.91), heart disease (crude HR = 3.96, 95% CI: 3.60–4.37), atrial fibrillation (crude HR = 5.81, 95% CI: 4.35–7.75), PVD (crude HR = 2.55, 95% CI: 2.14–3.04), hyperlipidemia (crude HR = 2.34, 95% CI: 2.02–2.70), cancer (crude HR = 2.10, 95% CI:1.66–2.65), and income. (Table 3) In stratified analyses, PUD appeared to be a risk factor for IS in all strata defined by age, sex, and duration of follow-up. (Table 2)

**FIGURE 2:**
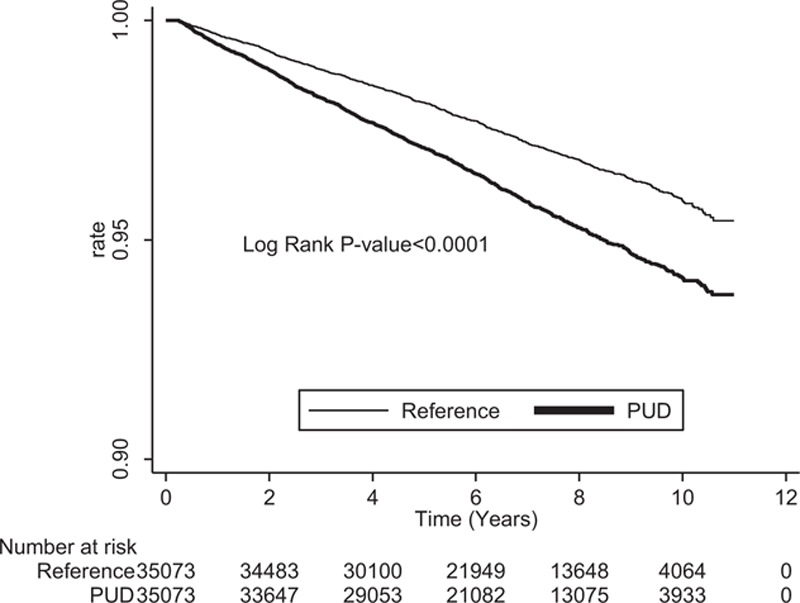
Stroke-free survival rates in patients with peptic ulcer disease (PUD) and the reference group from 1997 to 2011.

After adjusting for other risk factors, we found PUD was an independent risk factor for IS, with an adjusted HR (AHR) of 1.31 (95% CI: 1.20–1.41). The Kaplan-Meier survival analysis also showed a difference in the incidence rates of IS between the PUD and the references groups (*P* < 0.001 for the log-rank test) (Figure 2). Other independent risk factors included male sex (AHR = 1.23, 95% CI: 1.13–1.34), DM (AHR = 1.79, 95% CI: 1.63–1.97), hypertension (AHR = 2.02, 95% CI: 1.84–2.21), heart disease (AHR = 1.20, 95% CI: 1.07–1.33), atrial fibrillation (AHR = 1.44, 95% CI: 1.07–1.95), and PVD (AHR = 1.30, 95% CI: 1.09–1.23) (Table 3). Older age and lower income were also independent risk factors for IS, and both had a dose–response relationship with IS. After stratification, the risk of IS was higher in women (AHR = 1.32, 95% CI: 1.15–1.51) than men (AHR = 1.30, 95% CI: 1.17–1.44) (Table 4).

**TABLE 4:**
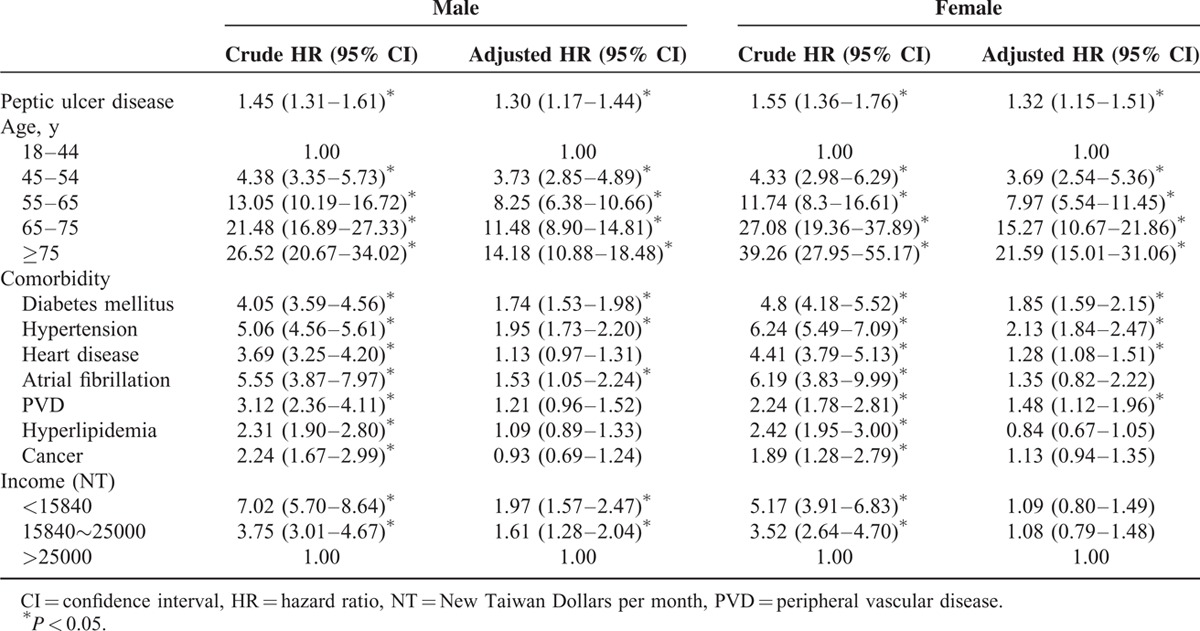
Crude and Adjusted HRs for the Development of Ischemic Stroke Stratified by Sex

## DISCUSSION

In this study, we found that PUD patients had a higher risk of developing IS, whereas they also had higher income and prevalence of hypertension, DM, heart disease, and hyperlipidemia. After adjusting for sex, age, income, and co-morbidity of hypertension, DM, and heart disease, PUD appeared to be an independent risk factor for IS, and this finding is compatible with the finding of a Swedish study that gastric ulcer was an independent risk factor for stroke.^17^

Psychological stress is a plausible etiology of the association between PUD and IS, and this is supported by some studies on stress and CVD. The Caerphilly Cohort Study evaluated the effects of psychological stress on IS and found a relative risk of 1.45.^18^ The risk was significantly increased in fatal case but not in nonfatal cases. The Health Survey for England studied psychological stress in an 8-year follow-up and found an increase in CVD deaths with an HR of 1.66.^19^ A case–control study in Spain found that stressful habits and type A behavior were associated with high risks of stroke,^20^ and the Finnish Public Sector Study also found that women workers with stress from low socioeconomic position had an increased risk of CVD.^21^ The effects of psychological stress had little relation to conventional risk factors in these studies.

There are several possible mechanisms of stress-induced vascular diseases. A case–control study revealed that platelet activation by increasing monocyte/leukocyte platelet aggregates was associated with psychological stress from low socioeconomic status.^22^ Activated platelets can be the first blood cells to adhere to the intact endothelial monolayer, even in the absence of endothelial disruption.^23^ This aggregation effect interacts with atherosclerotic lesions by delivering platelet-derived chemokines chemokine (C-C motif) ligand 5 (regulated on activation, normal T-cell expressed and secreted), and platelet factor 4 to the monocytes and endothelium of atherosclerotic arteries. Activated platelets also promote leukocyte binding of vascular cell adhesion molecule-1 and P-selectin and increase platelet adhesion to atherosclerotic endothelium.^24^ Circulatory proinflammatory cytokines including C-reactive protein, interleukin-6, and interleukin-1 increase in correlation with activated leukocyte aggregation.^25^ These phenomena in persons under stress would cause the formation and progression of atherosclerosis and subsequently provoke CAD and CVD development.

Under acute and chronic stress, the protective effects of the neuroendocrine system's hypothalamic-pituitary-adrenal (HPA) axis in response to internal and external stress^26^ are dysregulated. As stress develops, the levels of circulatory catecholamines, corticotropins, and cortisol increase, which results in elevated heart rate and blood pressure, increased hemostatic factors and blood viscosity,^27^ and impaired endothelial function.^28^ The sympathetic activity also increases the levels of interleukin-6 and C-reactive protein.^29^ These effects from dysregulated HPA axis may cause atherosclerosis.

Although only 20% of HP-infected patients develop duodenal ulcer,^30^ HP infection no doubt plays a major role in the development of PUD. There are also some evidence of the association between HP infection and stroke. Pietroiusti et al evaluated the seroprevalence of HP infection and found that it was 71.0% in atherosclerotic stroke patients and 70.2% in healthy participants.^31^ Whereas the differences did not reach statistical significance, there was higher prevalence of cytotoxin-associated gene-A (CagA) HP strain (42.8%) in atherosclerotic stroke patients, with an odds ratio of 3.04 compared with cardiogenic embolic stroke patients and an odds ratio of 4.3 compared with healthy participants. The findings suggested that CagA-positive HP strain infection was associated with atherosclerotic stroke. This strongly virulent strain infection was further demonstrated to have dose–response relations to both intima-media thickness and risk of developing atherosclerosis.^32^ Furthermore, Diomedi et al^33^ conducted a cohort study that enrolled new-onset IS patients with seropositive HP infection and found that CagA-positive patients had a higher risk of having stroke than CagA-negative patients, with a HR of 3.5. All these evidence in the literature support our finding Study of Women's Health Across the Nation that patients with PUD have a higher risk of developing IS.

There are some limitations in our study. The NHI mandates positive PUD findings from panendoscopy for the reimbursement of PUD medication, and therefore all the PUD cases in our study were confirmed by panendoscopy. Although this ensured the accuracy of diagnosis, PUD patients who did not receive panendoscopy or PPI/H2 blocker medication treatments were not covered in our analyses. This exclusion would misclassify PUD case into control group to be a differential misclassification and attenuate the effect. Although exclusion of those patients might limit the generalization of the study results, because panendoscopy rarely causes IS and was prescribed before the occurrence of IS in our study, the exclusion was unlikely to introduce remarkable bias to the study results. In addition, our study did not cover patients with very mild or asymptomatic IS who did not seek for medical care, and this might also affect the generalization of our findings. One of the most obvious strengths of our study is the large sample size, which is needed to evaluate the effects of many risk factors at the same time. Owing to the limitation of the information included in the LHID2000, however, we were unable to adjust for other personal risk factors for IS such as exercise and body mass index, smoking, diet habit, and drinking. Nonetheless, since both exercise and body mass index are closely related to the diseases that were adjusted in our final model, including DM, hypertension, and heart disease, we believe the residual confounding effects of these personal risk factors should be small, if existed. Another potential confounder that our study did not control is HP infection. Nonetheless, although PUD is now considered an infectious disease associated with HP,^12^ only a portion of people with HP infection develop PUD, and it was estimated that 30% to 65% of PUD patients were related to psychosocial stress.^13^ In addition to stress, smoking is an important common risk factors shared by PUD and IS. However, the prevalence of smokers in Taiwanese women is very low; for example, the prevalence in women aged 18 years or older was 3.9% in the national survey in 2001.^34^ When we analyzed the data on women separately, PUD remained an independent risk factor for IS with higher risk than men. Therefore, the possible confounding effect of smoking is very small and unlikely to affect our conclusion of an association between PUD and subsequent IS. Some PUD patients might receive antiplatelet medication after the diagnosis of PUD, which might attenuate the effect of PUD on developing IS and lead to underestimation of the risk of developing IS in PUD patients. Still, this would not affect the conclusions of our study. Further studies that can control those factors and verify our findings to other populations are needed to confirm and generalize our findings.

## CONCLUSIONS

By analyzing NHIRD in Taiwan, we found that patients with PUD had a higher risk of developing IS. The increase in risk was independent of conventional risk factors including older age, male sex, DM, hypertension, heart disease, and income, which were also found to be significant predictors of IS in the analyses. This association may arise from the fact that both psychological stress and HP infection are correlated with atherosclerosis. Managing PUD may be a promising method for reducing the risk of developing IS, especially in those without conventional risk factors.
